# ARIMA-Based Prediction of Heavy Metal Health Risks in Drinking Water Sources of Southwest Guizhou, China

**DOI:** 10.3390/toxics14020166

**Published:** 2026-02-11

**Authors:** Haihe Wang, Lin Zhong, Yuanyuan Sun, Qiuhua Li

**Affiliations:** 1 School of Mathematical Sciences, Guizhou Normal University, Guiyang 550014, China; wanghh@gznu.edu.cn; 2 Guizhou Ecological and Environment Monitoring Center, Guiyang 550081, China; 3 School of Life Sciences, Guizhou Normal University, Guiyang 550001, China; zhonglin@gznu.edu.cn; 4 Guizhou Key Laboratory of Advanced Computing, School of Cyber Science and Technology, Guizhou Normal University, Guiyang 550001, China; 5 Guizhou International Science & Technology Cooperative Base of Aquatic Ecology Research, Guiyang 550001, China

**Keywords:** Cr(VI), non-carcinogenic risk, age-specific risk, time-series forecasting, ARIMA, karst hydrogeochemistry

## Abstract

Drinking water represents a major pathway for human exposure to heavy metals, which are associated with long-term and cumulative health risks. This study investigated eight heavy metals (Ba, V, Cr(VI), Mn, Mo, Ni, Cu, Fe) in three centralized drinking water sources in southwest Guizhou from January 2021 to December 2023. Overall, the measured heavy metal concentrations complied with relevant national standards; however, transient exceedances of the Class I surface water standard were observed for Cu and Cr(VI) in certain months, with the most pronounced deviations occurring in 2022. Significant temporal variations were observed for Mn, Ni, Cu, and Fe, likely influenced by anthropogenic activities such as sewage discharge and agriculture, while Cr(VI), Mo, Ba, and V showed notable spatial differences linked to the region’s geological features. Health risk assessments showed that the non-carcinogenic risks for both adults and children remained below the maximum acceptable level of 5.0 × 10^−5^ a^−1^, with Cu, Mo, Ba, and V contributing most to the risks. In contrast, the carcinogenic risk associated with Cr(VI) exceeded the ICRP-recommended benchmark of 1.0 × 10^−6^ a^−1^, with estimated risks for children approximately 1.6 times higher than those for adults. In 2022, the carcinogenic risk was highest across all sources. Autoregressive integrated moving average (ARIMA) based forecasts suggest that Cr(VI)-related carcinogenic risk is likely to remain above acceptable levels in the near future, with an increasing trend projected for Mulanghe Reservoir, a declining trend for Xingxihu Reservoir, and relatively stable conditions for Weishanhu Reservoir. These findings underscore the need for targeted interventions, particularly in controlling Cr(VI) concentrations and protecting vulnerable populations.

## 1. Introduction

Heavy metals have characteristics such as potentiality, concealment, stability, irregularity, and bioaccumulation [1,2,3] and are widely present in natural environments, including water, soil, air, and sediments. They are both essential trace elements for the human body and may pose a direct or indirect threat to human health. Numerous scholars [4,5,6,7,8] have conducted in-depth studies on the behavior of heavy metals in these environments from multiple aspects, including analytical assessment, spatiotemporal variation, and migration transformation, providing crucial theoretical and technical support for the scientific management of local water resources.

The main sources of heavy metals in water are natural and anthropogenic [9]. Natural sources include processes such as geological erosion, natural weathering, and desertification [10], while anthropogenic activities encompass mining operations, heavy metal smelting, industrial wastewater discharge, agricultural planting, domestic waste disposal, sewage discharge [11,12], fossil fuel burning, etc. [13,14,15,16]. These heavy metals enter the human body through drinking water, accumulate within the body, and pose a threat to human health beyond certain thresholds [17,18,19], especially to the liver, kidneys, digestive system, and nervous system [20]. Long-term exposure to heavy metals may also increase the risk of cancer [21,22,23,24,25,26].

In recent years, most scholars have begun to study heavy metals in surface water in the Yunnan-Guizhou Plateau region, including the distribution and ecological risks of heavy metals in the plateau lake Caohai [27,28], manganese mining areas, and coal mining areas [29,30]. Research on heavy metal pollution and risk assessment, as well as on heavy metal distribution and health risks in drinking water sources [31,32], has been conducted. Analysis of the spatiotemporal trends of heavy metals in drinking water, conducting health risk assessments and predictions, and examining pollution status and source analysis can reflect the drinking water safety status of drinking water sources and, at the same time, provide a scientific basis and main decision-making objectives for environmental risk management.

However, most existing studies in the Yunnan–Guizhou Plateau have concentrated on plateau lakes and major river basins, whereas long-term, high-frequency investigations of heavy metals in centralized, city-scale drinking-water sources remain relatively scarce. In addition, prior research has largely focused on concentration assessment or single-time-point risk evaluation, while integrated frameworks that jointly incorporate long-term monitoring, age-specific health risk assessment, and time-series forecasting are still limited.

Xingyi City is located in the southeast of the Yunnan-Guizhou Plateau, at the junction of Yunnan, Guizhou, and Guangxi provinces, and is rich in mineral resources, mainly including more than 10 kinds of minerals such as Au, Fe, coal, and Mo. In recent years, many scholars have investigated heavy metals in surface water at the source of the Pearl River, including the Huangni River, Nanpan River, Beipan River, and other river basins, and found that there are varying degrees of enrichment of heavy metals in water bodies or sediments [2], posing varying degrees of health risks to humans. However, there are relatively few studies on heavy metals in centralized drinking water sources in Xingyi City.

Therefore, there is a clear knowledge gap regarding (i) the long-term temporal variability and spatial heterogeneity of trace heavy metals among centralized drinkin water sources in Xingyi City, (ii) the extent to which chronic exposure may impose non-carcinogenic and carcinogenic risks to sensitive populations (adults vs. children), and (iii) whether future health-risk trends can be reliably predicted to inform risk early warning and proactive management.

Based on this, this paper selects three drinking water sources in Xingyi City as the research objects to analyze the mass concentration and spatiotemporal distribution characteristics of eight heavy metals (Ba, V, Cr(VI), Mn, Mo, Ni, Cu, Fe) in water bodies. Using an environmental health risk assessment model, the potential carcinogenic and non-carcinogenic health risks of drinking water sources to different age groups (adults and children) were evaluated. Concurrently, the ARIMA prediction model constructed using IBM SPSS Statistics 26.0 (IBM Corp., Armonk, NY, USA) software was used to predict and analyze future heavy metal health risks. The study aims to provide theoretical support for local ecological environment management in heavy metal control, early warning, and forecasting, and to offer a reference for the health risk assessment of other environmental elements.

Specifically, this study aims to address the following scientific questions: (1) What are the long-term temporal trends and seasonal patterns of the detected heavy metals in the three drinking water sources? (2) Which metals dominate the non-carcinogenic risks and how do the risks differ between adults and children? (3) Under local hydrogeochemical conditions, is the carcinogenic risk primarily driven by Cr(VI)? (4) Can ARIMA-based time-series modeling provide reliable short-term forecasts of health risks for early warning?

This study contributes to the existing literature in three main aspects: (i) it provides a three-year (2021–2023) monthly monitoring dataset of trace heavy metals from three centralized drinking-water sources in Xingyi City, a mining-influenced plateau region where such long-term data remain limited; (ii) integrating long-term monitoring with spatiotemporal analysis and age-specific carcinogenic and non-carcinogenic health risk assessment to identify priority metals and sensitive populations; and (iii) applying ARIMA-based time-series forecasting to predict future carcinogenic risk trends of Cr(VI), thereby providing a quantitative basis for risk early warning and targeted management of drinking-water safety.

## 2. Materials and Methods

### 2.1. Study Area

The Mabie River is a first-level tributary on the left bank of the Nanpan River. It originates in Panzhou City, Guizhou Province, flows through the urban area of Xingyi City, and joins the Nanpan River at Wanfeng Lake in Anlong County. The precipitation basin has an average annual precipitation of 1340 mm and an average annual runoff of 1.65 billion cubic meters. The water system spreads out in a tree-like pattern, with 18 first-level tributaries. Among them, the largest tributary in Xingyi City is the Mulang River, followed by the Nasheng River. There are three centralized drinking water sources in Xingyi City, namely the Mulanghe Reservoir, Xingxihu Reservoir, and Weishanhu Reservoir, which are located on the first-level tributaries of the Mabie River: the Mulang River, Nasheng River, and Nahui River, respectively. All three drinking water sources are medium-sized reservoirs built by humans. In 2022, they served 770,000 people in the city, with an average annual water supply of 136.36 million tons.

### 2.2. Sampling Site Layout and Sampling Analysis

**Figure 1:**
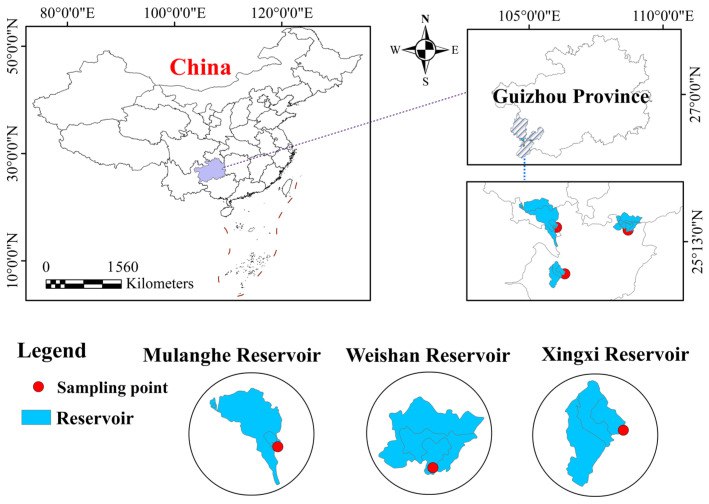
Sampling site schematic diagram of the study area.

The layout of sampling points at the water intake points of the three central urban drinking water sources in Xingyi City is shown in Figure 1. The sampling points were all selected near the water intake points of the waterworks pump rooms of the drinking water sources, and the water samples were more representative.

From January 2021 to December 2023, sampling was conducted at fixed points once a month (a total of 36 sampling campaigns). Three centralized drinking water sources were monitored, yielding 108 sets of routine monitoring data (36 months × 3 sources). The sampling procedures were carried out in accordance with the Technical Specifications for Surface Water Environmental Quality Monitoring (HJ 91.2-2022) [33]. The sampling technical requirements were implemented following the “Water Quality—Guidance on Sampling Techniques” (HJ 494-2009) [34], and the collection, preservation, and handling of water samples were performed according to the “Water Quality Sampling—Technical Regulation of the Preservation and Handling of Samples” (HJ 493-2009) [35].

Cu, Fe, Mn, Mo, Ni, Ba, and V were immediately filtered through 0.45 μm microporous membranes after on-site sampling and stored in sealed polyethylene bottles at 4 °C until analysis. The determination was carried out using inductively coupled plasma–mass spectrometry (ICP–MS; Agilent 7800 ICP–MS, Agilent Technologies, Santa Clara, CA, USA) following HJ 700-2014 [36].

Cr(VI) was determined without pretreatment after sampling using the 1,5-diphenylcarbohydrazide UV–Vis spectrophotometric method (GB 7467-87) [37] with a UV–Vis spectrophotometer (Shanghai Jinghua Technology Instrument Co., Ltd., Shanghai, China).

As, Hg, and Se were determined using atomic fluorescence spectrometry (AFS) according to HJ 694-2014 (atomic fluorescence spectrophotometer, Beijing Jitian Instrument Co., Ltd., Beijing, China).

Quality assurance and quality control (QA/QC) were conducted in accordance with the “Technical Specifications for Surface Water Environmental Quality Monitoring” (HJ/T 91.2-2022) [33] and the “Quality Assurance Manual for Environmental Water Quality Monitoring” (2nd Edition).

Field-coded samples, full-procedure blanks, and parallel samples were included during sampling and analysis. Duplicate (parallel) samples were analyzed at a frequency of one per ten routine samples (10%). For ICP–MS analysis, multi-point external calibration was performed using certified mixed-element standards, internal standards were applied to correct for instrumental drift and matrix effects, and continuing calibration verification (CCV) was conducted periodically. For UV–Vis and AFS determinations, batch calibration curves were established and verified using quality control standards. The recovery rates and precision indicators met the acceptance criteria specified by the relevant methods.

The method detection limits (MDLs) of the target metals/metalloids are summarized in Table 1, and the analytical methods, instruments, and QA/QC procedures are presented in Table 2.

In total, 65 elements were analyzed in this study, including 19 heavy metals/metalloids (Cu, Zn, Se, As, Hg, Cd, Cr(VI), Pb, Fe, Mn, Mo, Co, Be, B, Sb, Ni, Ba, V and Tl). During the monitoring period, only Ba, V, Cr(VI), Mn, Mo, Ni, Cu, and Fe were detected, whereas the remaining metals/metalloids (including Pb, Cd, and As) were consistently below the MDLs (Table 1) and therefore were excluded from the quantitative health-risk assessment to avoid uncertainties associated with non-detect substitution. As a result, Cr(VI) was the only carcinogenic contaminant consistently detected above the method detection limit. Accordingly, total carcinogenic risk was quantified solely on the basis of Cr(VI) and should therefore be interpreted as a conservative estimate.

### 2.3. Water Environment Health Risk Assessment Model

The most significant and risky exposure route for health risks is direct drinking [38]. Therefore, this study mainly analyzed the health risks caused by the direct drinking route to different populations (adults and children) and conducted the research using the carcinogenic and non-carcinogenic health risk assessment models recommended by the United States Environmental Protection Agency (US EPA) [39,40,41,42,43,44]. Specific evaluation model formulas are shown in (1) to (5).

#### 2.3.1. Carcinogenic Health Risk Assessment Model


(1) $$R_{c}=\sum_{i=1}^{k}R_{i}^{c}$$

(2) $$R_{i}^{c}=\left[1-\exp\left(-D_{i}Q_{i}\right)\right]/Y$$

*D_i_* = *Mρ_i_*/*m* (3)

In the formula, $R_{i}^{c}$ represents the average annual health risk (a^−1^) of one of the evaluated carcinogenic heavy metals (total of k) through drinking water;

$R_{c}$ represents the total health hazard (a^−1^) of all the evaluated carcinogenic heavy metals;

*D_i_* is the average daily exposure dose per unit body weight (mg·kg^−1^·d^−1^) of a certain evaluated carcinogenic heavy metal produced through the drinking water route;

$Q_{i}$ is the carcinogenic slope factor of the heavy metal through drinking water (kg·d·mg^−1^);

Y represents the average life expectancy of residents. According to the data released by the Bureau of Statistics of Guizhou Province in 2012, the average life expectancy of residents in Guizhou was 74.86 years [45];

*M* represents the average daily water intake for different populations. According to the Highlights of the Chinese Exposure Factors Handbook (Adults) and (Children: 6–17 Years) compiled by the Ministry of Environmental Protection, the average daily water intake for adults is 1.478 L·d^−1^ [18] (pp. 87–91). The average daily water intake for children was 1.053 L·d^−1^ (for 6–9 years) [45] (pp. 60–67);

*ρ_i_* is the mass concentration of a heavy metal under evaluation, mg·L^−1^;

*m* represents the average body weight of different populations. According to the Highlights of the Chinese Exposure Factors Handbook (Adults) and (Children: 6–17 Years) compiled by the Ministry of Environmental Protection, the average body weight of adults in the urban population of Guizhou is 59.2 kg [18] (pp. 759–763). The average weight of children was 26.5 kg (for 6–9 years) [45] (pp. 854–861).

#### 2.3.2. Non-Carcinogenic Health Risk Assessment Model


(4) $$R_{n}=\sum_{i=1}^{m}R_{i}^{n}$$

(5) $${R_{i}}^{n}=(D_{i}/RFD_{i})\times 10^{-6}/Y$$

In the formula, $R_{n}$ represents the total risk (a^−1^) of health hazards of non-carcinogenic heavy metals evaluated;

$R_{i}^{n}$ represents the average annual health risk (a^−1^) of a non-carcinogenic heavy metal through the drinking water route;

*D_i_* represents the average daily exposure dose per unit body weight (mg·kg^−1^·d^−1^) of a non-carcinogenic heavy metal under evaluation produced through drinking water.

*Y* represents the average life expectancy of residents, the same as above, with a value of 74.86 years [46];

$RFD_{i}$ It is the reference dose (mg·kg^−1^·d^−1^) of a non-carcinogenic heavy metal ingested by the human body through drinking water.

#### 2.3.3. Determination of Parameters

According to the classification system developed by the International Agency for Research on Cancer (IARC) and the World Health Organization (WHO), Cr(VI) is a carcinogenic heavy metal, while Cu, Fe, Mn, Mo, Ni, Ba, and V are non-carcinogenic heavy metals. The carcinogenic intensity coefficients and reference doses of non-carcinogenic substances in their models were selected as shown in Table 3 [7,38,47].

### 2.4. ARIMA Time Series Prediction Model

The ARIMA (autoregressive integrated moving average) modeling and forecasting were implemented using IBM SPSS Statistics 26.0 (IBM Corp., Armonk, NY, USA). Stationarity of the time-series data was first examined using the Augmented Dickey–Fuller (ADF) test, and the differencing order (d) was determined accordingly. The autoregressive and moving-average orders (p and q) were preliminarily identified based on the autocorrelation function (ACF) and partial autocorrelation function (PACF) plots (95% confidence interval; ±2 standard deviations). Model adequacy was then evaluated by residual diagnostics using the residual ACF/PACF and the Ljung–Box Q statistics. A model was considered acceptable when the residual autocorrelations fell within the 95% confidence interval and the Q-statistic *p*-values (e.g., Q6) were >0.05, indicating that the residuals behaved as white noise. Based on these criteria, the final ARIMA structures were determined as ARIMA(2,0,3) for Weishanhu Reservoir, ARIMA(5,1,5) for Xingxihu Reservoir, and ARIMA(2,1,2) for Mulanghe Reservoir.

## 3. Results and Discussion

### 3.1. Statistical Analysis of Heavy Metals in Xingyi City’s Drinking Water Sources

**Figure 2:**
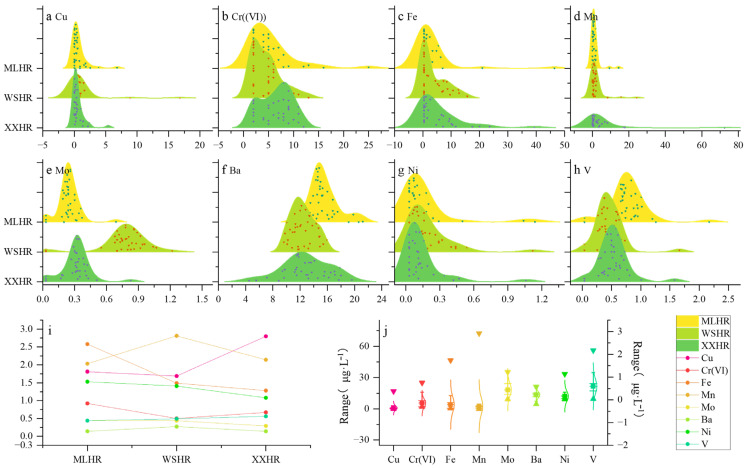
Ridge plots and coefficient of variation (CV) plot and Box plot of the concentration range distribution for heavy metals in the study area. Subplots (**a**–**h**) are ridge plots showing the concentration distributions (unit: μg·L^−1^) of elements (Cu, Cr(VI), Fe, Mn, Mo, Ba, Ni, V) in the study area: MLHR, WSHR, and XXHR. Subplot (**i**) is a line plot presenting the variation trend of the coefficient of variation (CV) for the above elements across MLHR, WSHR, and XXHR groups. Subplot (**j**) is a Box plot, the primary *Y*-axis (left) applies to elements Cu, Cr(VI), Fe, Mn, and Ba, while the secondary *Y*-axis (right) corresponds to elements Mo, Ni, and V. MLHR = Mulanghe Reservoir; WSHR = Weishanhu Reservoir; XXHR = Xingxihu Reservoir.

The monitoring and statistical results of heavy metals in the water bodies of the drinking water sources of three central cities in Xingyi City within the study area are shown in Figure 2. During the monitoring period, the mass concentration of Mn varied the most, ranging from 0.12 μg·L^−1^ to 72.4 μg·L^−1^, and the mass concentration of Mo varied the least, ranging from 0.06 μg·L^−1^ to 1.22 μg·L^−1^. The average mass concentrations of heavy metals, from largest to smallest, were Mn > Fe > Ba > Cr(VI) > Cu > V > Ni > Mo. The maximum monthly average concentrations of Cu and Cr(VI) were 16.8 μg·L^−1^ and 25.0 μg·L^−1^, respectively, both higher than the Class I standard limit, while their average values were lower than the standard limit, according to the standard limits of heavy metal concentrations in the “Environmental Quality Standards for Surface Water” (GB 3838-2002) [48]. The concentrations of Cu and Cr(VI) exceeded the Class I standard in some months during the data monitoring period. The maximum mass concentrations of six heavy metals, namely Mn, Fe, Ba, V, Ni, and Mo, were all below the limits of the “Standard for Drinking Water” (GB 5749-2006) [49], and there were no months with excessive concentrations. As mentioned earlier, heavy metals are accumulative and have cumulative effects [1,2]. Therefore, further analysis and evaluation of the health risk level of heavy metals are needed.

### 3.2. Spatiotemporal Distribution Characteristics of Heavy Metal Concentrations

#### 3.2.1. Temporal Variation Characteristics

The coefficient of variation can reflect the degree of temporal dispersion of the same heavy metal concentration at the same monitoring point. Figure 2 shows the temporal distribution characteristics of heavy metal mass concentrations in the study area. When conducting the analysis of temporal dimension variation characteristics, if the coefficient of variation is less than 0.1, which is lower than the coefficient of variation of 0.1 for the heavy metal determination method itself, it is considered weak variability, indicating that there is no significant temporal difference in heavy metal concentrations within the study area. A coefficient of variation between 0.1 and 1 is considered moderate variability. The coefficients of variation in four heavy metals in the study area—Cr(VI), Mo, Ba, and V—are all within this range, indicating a certain degree of variation in the heavy metal concentrations in the study area over time. The coefficient of variation is greater than 1 [50], and the coefficients of variation in the four heavy metals—Cu, Fe, Mn, and Ni—in the study area are all higher than 1, indicating significant temporal differences in heavy metal concentrations in the study area, as shown in Figure 2i.

The pronounced temporal variability observed for Cu, Fe, Mn, and Ni may be associated with temporal changes in external inputs acting at the watershed scale during the monitoring period. Although direct source measurements were not conducted in this study, the observed temporal fluctuations were likely influenced by anthropogenic activities, such as domestic sewage discharge and agricultural non-point source runoff, which can vary seasonally and affect metal levels in drinking-water sources. Therefore, this interpretation is presented as a plausible explanation rather than a confirmed source attribution [1,51].

#### 3.2.2. Characteristics of Spatial Variation

**Figure 3:**
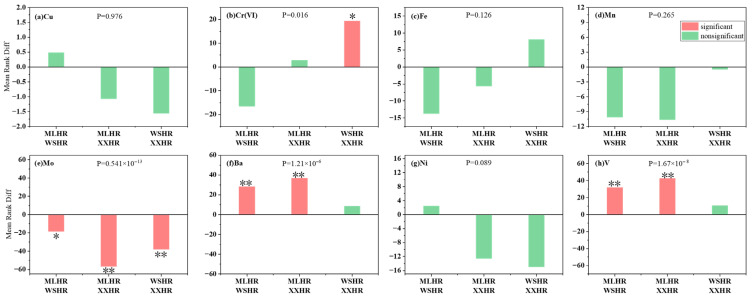
Paired Comparison Plot of heavy metals in the study area. This figure displays the mean rank differences of 8 element concentrations (Cu, Cr(VI), Fe, Mn, Mo, Ba, Ni, V) under three drinking water sources (MLHR, WSHR, XXHR), tested by the Kruskal–Wallis ANOVA (K-W ANOVA, a non-parametric test for comparing multiple independent groups). *p* ≥ 0.05: The samples come from the same population, the populations are not significantly different. *p* < 0.05: The samples come from different populations, the populations are significantly different * = *p* < 0.05 (statistically significant between two drinking water sources), ** = *p* < 0.01 (highly statistically significant between two drinking water sources). MLHR = Mulanghe Reservoir; WSHR = Weishanhu Reservoir; XXHR = Xingxihu Reservoir.

Spatial variation in heavy metal concentrations across the study area from 2021 to 2023 was analyzed using OriginPro 2026 with the non-parametric Kruskal–Wallis analysis of variance. The spatial distribution and statistical results are presented in Figure 3.

As shown in Figure 3, Cr(VI), Mo, Ba, and V exhibited significant spatial variation among the three reservoirs, with *p* values of 0.016, <0.01, <0.01, and <0.01, respectively (all *p* < 0.05). In contrast, Cu, Fe, Mn, and Ni showed no significant spatial differences, with *p* values of 0.98, 0.13, 0.26, and 0.09, respectively (all *p* > 0.05).

The results of Dunn’s multiple comparison test are shown in Figure 3b,e,f,h. Cr(VI) showed a significant difference between Weishanhu Reservoir and Xingxihu Reservoir. Ba and V exhibited significant differences between Mulanghe Reservoir and Weishanhu Reservoir, as well as between Mulanghe Reservoir and Xingxihu Reservoir. Mo showed significant differences among all three reservoirs, including Mulanghe Reservoir versus Weishanhu Reservoir, Mulanghe Reservoir versus Xingxihu Reservoir, and Weishanhu Reservoir versus Xingxihu Reservoir.

As shown in Figure 3a,c,d,g, Dunn’s multiple comparison test indicated that Cu, Fe, Mn, and Ni did not exhibit significant differences between any pair of reservoirs, including Mulanghe Reservoir, Weishanhu Reservoir, and Xingxihu Reservoir.

Based on the statistically significant temporal and spatial patterns identified in Section 3.2, further discussion is provided below to explore the potential factors underlying these variations.

The combined analysis of temporal and spatial variation provides insights into the potential factors controlling heavy metal concentrations in the study area. Cu, Fe, Mn, and Ni exhibited significant temporal variation but no significant spatial differences among the three reservoirs, suggesting that their concentrations may be influenced by time-dependent external inputs acting at the watershed scale. Such inputs may include seasonal or interannual variations in human activities, hydrological conditions, or diffuse runoff processes.

In contrast, Cr(VI), Mo, Ba, and V showed pronounced spatial heterogeneity while remaining temporally stable during the monitoring period. This spatial pattern is consistent with the influence of site-specific environmental controls, such as local hydrogeochemical conditions and water–rock interactions, such as local hydrogeochemical conditions, water–rock interactions, and differences in geological background among reservoirs, particularly in karst-influenced catchments.

It should be noted that these interpretations are based on statistical patterns rather than direct source identification. In the absence of dedicated geochemical source apportionment, the proposed explanations should be regarded as plausible inferences that contextualize the observed trends, rather than definitive conclusions regarding contaminant sources.

### 3.3. Health Risk Assessment of Heavy Metals in Drinking Water Sources in the Study Area

**Figure 4:**
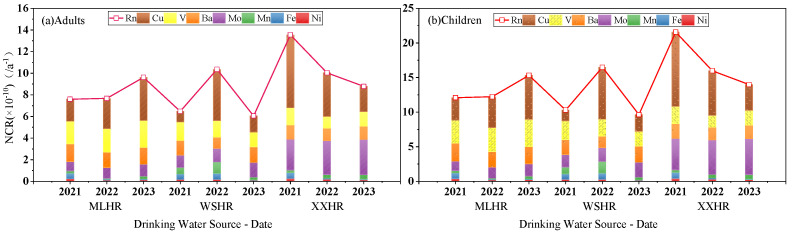
Stacked Bar Chart of NCR for Adults and Children under Different Drinking Water Sources and Years. The drinking water sources in the figure include MLHR, WSHR, and XXHR, with data covering the years 2021–2023; each colored block in the stacked bar chart represents the non-carcinogenic risk values of the corresponding elements (Cu, V, Ba, Mo, Mn, Fe, Ni) as well as the comprehensive non-carcinogenic risk value. NCR = Non-Carcinogenic Risk; MLHR = Mulanghe Reservoir; WSHR = Weishanhu Reservoir; XXHR = Xingxihu Reservoir.

Based on the health risk assessment model and parameters recommended by the United States Environmental Protection Agency (US EPA), calculate the carcinogenic risk (for adults and children) of Cr(VI) and the non-carcinogenic risk (for adults and children) of Cu, Fe, Mn, Ni, Mo, Ba, and V in three drinking water sources (Mulanghe Reservoir, Weishanhu Reservoir, and Xingxihu Reservoir) within the study area. The combined non-carcinogenic risk is the cumulative sum of the health risk assessment results corresponding to different heavy metals, as shown in Figure 4.

#### 3.3.1. Non-Carcinogenic Risk Assessment

Overall, the non-carcinogenic risk index and the combined non-carcinogenic risk index from a single heavy metal in drinking water within the study area were both within the safe range and below the maximum acceptable risk level of 5.0 × 10^−5^ a^−1^ recommended by the International Commission on Radiological Protection (ICRP). The concentrations of heavy metals (Cu, Fe, Mn, Ni, Mo, Ba, V) in drinking water has no significant health impact on the human body. Due to the large amount of data on the non-carcinogenic risk of heavy metals, this paper further statistically analyzed the non-carcinogenic risk of heavy metals in the study area, and the evaluation results are shown in Figure 4.

In terms of individual heavy metal concentrations, among the seven heavy metal concentrations evaluated, Cu has the highest non-carcinogenic index, while Ni has the lowest. As shown in Figure 4, the overall average non-carcinogenic risk of different types of heavy metals in adults and children is ranked as follows: Cu > Mo > V > Ba > Mn > Fe > Ni. The non-carcinogenic health risk assessment values of each indicator were well below the negligible levels recommended by the International Commission on Radiological Protection (ICRP) [52]. Since the non-carcinogenic risks of Fe, Mn, and Ni in adults and children are relatively low and not obvious in Figure 4, and the sum of Cu, Mo, Ba, and V accounts for more than 90% of the average annual non-carcinogenic health risk assessment, more attention should be paid to Cu, Mo, Ba, and V in Xingyi City’s drinking water non-carcinogenic factors.

In terms of different drinking water sources, among the three drinking water sources in the study area, Xingxihu Reservoir had the highest average annual comprehensive non-carcinogenic index: 10.8 × 10^−10^ a^−1^ for adults and 17.2 × 10^−10^ a^−1^ for children. Weishanhu Reservoir had the lowest average annual comprehensive non-carcinogenic index: 7.6 × 10^−10^ a^−1^ for adults and 12.1 × 10^−10^ a^−1^ for children. The overall non-carcinogenic risk assessment, from low to high, was Weishanhu Reservoir < Mulanghe Reservoir < Xingxihu Reservoir. The non-carcinogenic risk assessment values of the three drinking water sources are well below the negligible level recommended by the International Commission on Radiological Protection (ICRP), indicating that the health risks caused by non-carcinogenic factors are very small and will not pose a significant threat to local residents.

When analyzed over different years, the combined non-carcinogenic risk (for adults and children) of Mulanghe Reservoir showed an increasing trend year by year. The combined non-carcinogenic risk (for both adults and children) of Xingxihu Reservoir showed a decreasing trend year by year. The combined non-carcinogenic risk of Weishanhu Reservoir was higher in 2022 than in 2021 and 2023.

#### 3.3.2. Assessment of Carcinogenic Risk

**Figure 5:**
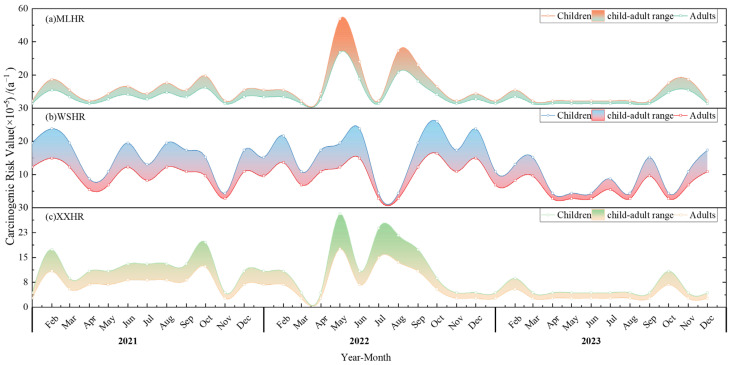
Temporal changes in carcinogenic risk among different groups (adults and children) and in different study regions. Subplots (**a**–**c**) correspond to the MLHR, WSHR, and XXHR, respectively. The filled area (labeled as ‘Children and Adult Risk Range’) represents the risk range between children and adult populations.

As shown in Figure 5. During the monitoring period, carcinogenic heavy metal concentrations such as Cd, As, and Co were not detected. This study only investigated Cr(VI) in drinking water for carcinogenic risk and did not investigate other carcinogenic factors (Cd, As, Co, etc.) in drinking water. Therefore, in this study, the carcinogenic risk caused by Cr(VI) was considered consistent with the overall carcinogenic risk.

In the study area, the ranges of carcinogenic risk caused by Cr(VI) in the water bodies of the three drinking water sources were as follows: Mulanghe Reservoir (adults 2.7–33 × 10^−5^ a^−1^, children 4.3–53 × 10^−5^ a^−1^), Weishanhu Reservoir (adults 2.7–16 × 10^−5^ a^−1^, children 4.3–25 × 10^−5^ a^−1^), Xingxihu Reservoir (adults 2.7–17 × 10^−5^ a^−1^, children 4.3–28 × 10^−5^ a^−1^). The highest adult carcinogenic risk assessment occurred at the Mulanghe Reservoir, with a Cr(VI) mass concentration of 0.025 mg/L in May 2022; the lowest carcinogenic risk assessment was 2.7 × 10^−5^ a^−1^, with no Cr(VI) detected. In this study, the carcinogenic risk in children was 1.6 times that in adults. Similar age-dependent differences have also been reported in previous studies, with children exhibiting approximately 1.1–2.5 fold higher carcinogenic risks than adults, see Table 4 [4,53,54,55,56].

The ranking of the average annual carcinogenic risk caused by Cr(VI) among the three drinking water sources for adults and children was Weishanhu Reservoir (adults: 8.8 × 10^−5^ a^−1^, children: 1.4 × 10^−4^ a^−1^) > Mulanghe Reservoir (adults: 7.2 × 10^−5^ a^−1^, children: 1.11 × 10^−4^ a^−1^) > Xingxihu Reservoir (adults: 6.1 × 10^−5^ a^−1^, children: 9.8 × 10^−5^ a^−1^). The trends of the average annual carcinogenic risk (for adults and children) at Mulanghe Reservoir, Xingxihu Reservoir, and Weishanhu Reservoir were largely the same, with the average annual carcinogenic risk in 2022 being higher than that in 2021 and 2023; the average annual carcinogenic risk was the lowest in 2023.

The average annual carcinogenic risk (for both adults and children) caused by ingested water in the three drinking water sources was higher than the negligible level recommended by the International Commission on Radiological Protection (ICRP) and also exceeded the maximum acceptable risk level of 1.0 × 10^−6^ a^−1^ recommended by the Swedish Environmental Protection Agency, the Royal Society of the United Kingdom, and the Dutch Ministry of Infrastructure and Water Management [52]. This indicates that Cr(VI) poses a health risk to both adults and children through drinking water intake. In future research and regulation, emphasis should be placed on the monitoring and assessment of Cr(VI), and Cr(VI) should be a priority control object for risk decision-making management. This study will further analyze and predict the carcinogenic risk level of Cr(VI).

### 3.4. Prediction of Carcinogenic Risk Based on ARIMA Model

In this study, the main health risk was the carcinogenic risk of Cr(VI) intake in both adults and children. The carcinogenic risk in children was higher than that in adults and 1.6 times that of adults. This study intends to use SPSS software and the ARIMA model to predict the carcinogenic risk among children in three drinking water sources within the study area.

#### 3.4.1. Data Stabilization Processing

To apply the ARIMA model, it is necessary to conduct a stationary analysis of the data first to determine whether the analyzed data has a significant changing trend. Data with a significant upward or downward trend is not suitable for direct prediction by the ARIMA model and needs to be further stabilized before prediction. The time series stationarity test was performed using the ADF test (unit root test) with EViews 14 (IHS Markit, Irvine, CA, USA) software [57,58,59] for stationarity analysis of the carcinogenic risk data of Cr(VI) intake in children at three drinking water sources, as shown in Table 5. The statistical values t of Mulanghe Reservoir and Xingxihu Reservoir were −2.0 and −2.2, respectively, both greater than the critical values of 1%, 5%, and 10%, and the *p*-values were 0.26 and 0.20, respectively, both greater than 0.05 and 0.01. Therefore, the null hypothesis—that the sequence is not stationary—cannot be rejected. The sequences of Mulanghe Reservoir and Xingxihu Reservoir were processed for first-order differential stabilization, with statistical values t of −8.4 and −10.7, respectively, both less than the critical values of 1%, 5%, and 10%, and *p*-values of 0.00 and 0.00, respectively, less than 0.05 and 0.01. The null hypothesis was rejected. The first-order differential sequences of the Mulanghe Reservoir and Xingxihu Reservoir are stationary sequences. For Weishanhu Reservoir, without differentiation, the statistical value t is −4.4, less than the critical values of 1%, 5%, and 10%, the *p*-value is <0.001, less than 0.05 and 0.01, and the null hypothesis is rejected. The original sequence of Weishanhu Reservoir is a stationary sequence, and the d values of Mulanghe Reservoir, Xingxihu Reservoir, and Weishanhu Reservoir are 1, 1, and 0, respectively.

#### 3.4.2. Model Determination

**Figure 6:**
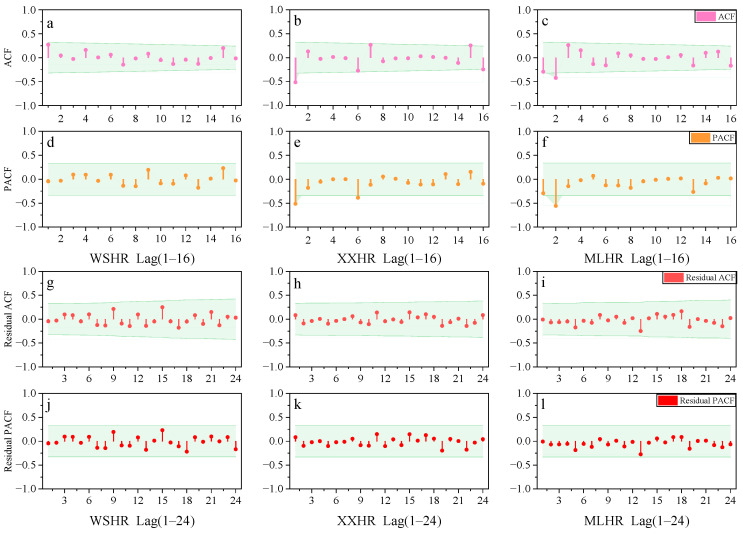
Function plots of Heavy Metal Comprehensive Carcinogenic Risk Prediction Model (ARIMA). The *Y*-axis represents the ACF, PACF, Residual ACF and Residual PACF (ranging from −1 to 1), with the shaded area indicating the 95% confidence interval. ACF (**a**–**c**) = Autocorrelation Coefficient, PACF (**d**–**f**) = Partial Autocorrelation Coefficient. Residual ACF (**g**–**i**) = Residual Autocorrelation Coefficient; Residual PACF (**j**–**l**) = Residual Partial Autocorrelation Coefficient.

The original sequence of Weishanhu Reservoir and the first-order difference sequences of Xingxihu Reservoir and Mulanghe Reservoir were analyzed using ACF plots and PACF plots, respectively, as shown in Figure 6. The *Y*-axis represents the ACF, PACF, Residual ACF, and Residual PACF (ranging from −1 to 1), with the shaded area indicating the 95% confidence interval. From Figure 6a,d, it can be seen that the correlation coefficient and partial correlation coefficient of the Weishanhu Reservoir both contract within two standard deviations, that is, both are within the 95% confidence interval. From Figure 6a, it can be seen that the correlation coefficient rapidly approaches 0 but does not reach 0 at the lag order 3, and then the lag order coefficient changes randomly, determining the q value as 3. From Figure 6d, it can be seen that the partial correlation coefficient quickly approaches 0 at the second lag order without reaching 0, and then the random variation in the lag order coefficient determines that the *p* value is 2. Therefore, it can be preliminarily determined that the prediction model of Weishanhu Reservoir is ARIMA (2,0,3). Likewise, as shown in Figure 6b,e, the correlation coefficient of Xingxihu Reservoir rapidly approaches 0 at the fifth lag, then the coefficient randomly changes to determine the q value as 5; the partial correlation coefficient rapidly approaches 0 at the fifth lag, then the coefficient randomly changes, determining the *p* value as 5. The prediction model of Xingxihu Reservoir is preliminarily determined to be ARIMA (5,1,5). In addition, different types of ACF plots and PACF plots can also determine the *p*-value and q-value of the model based on whether the autocorrelation coefficient and the partial correlation coefficient exceed the standard deviation limit (95% confidence interval) of two times. As shown in Figure 6c,f, the q value of the Mulanghe Reservoir can be determined as 2 when the autocorrelation coefficient of the Mulanghe Reservoir exceeds the standard deviation limit of two times at the second lag. The partial correlation coefficient also exceeds two times the standard deviation limit at the second lag order, and the *p*-value is determined to be 2, preliminarily determining the Mulanghe Reservoir prediction model as ARIMA (2,1,2).

As shown in Figure 6 and Table 6. To further verify the adaptability of the selected model, it is necessary to conduct white noise analysis on the residuals of the selected model through residual ACF and PACF plots and Q statistics, etc. [60]. If the sequence is not white noise, it indicates that the information extraction is incomplete and the model needs to be adjusted, From the ACF and PACF plots of the model residuals, it can be seen that the autocorrelation and partial correlation coefficients of the residuals of the three drinking water source models are all within the 95% confidence interval, and the residual sequences are stationary. From the results of the Q statistic, the *p*-values of Q6 are 0.64, 0.85, and 0.06, respectively, all greater than 0.05. The null hypothesis cannot be rejected at the significance level of 0.05. The residuals of the models have no autocorrelation and are white noise. The three models meet the requirements. Therefore, the selected model can predict the level of carcinogenic intake risk in children at the three drinking water sources.

**Figure 7:**
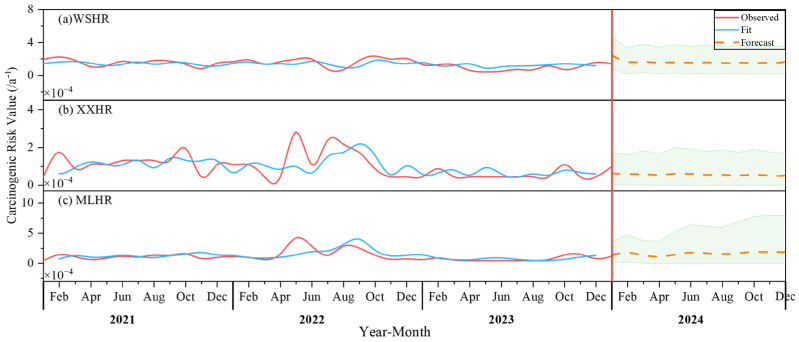
Carcinogenic risk prediction results of heavy metals in children in drinking water source. The filled parts are the 95% confidence intervals, and the predicted carcinogenic risk results for study area are all within the confidence interval. the red vertical line is used to distinguish between the observed values and the forecasted values, and the green background represents the confidence interval.

The results of the predicted carcinogenic risk levels for heavy metal intake among children at three drinking water sources—Weishanhu Reservoir, Xingxihu Reservoir, and Mulanghe Reservoir—based on the ARIMA (2,0,3), (5,1,5), and (2,1,2) models, respectively, are shown in Figure 7. The predicted sequences of the three drinking water sources show different changing trends. Using the ADF test for stationarity verification, it can be determined that the predicted sequence of Weishanhu Reservoir is a stationary sequence, while the predicted sequences of Xingxihu Reservoir and Mulanghe Reservoir are non-stationary sequences, as shown in the figure. The predicted sequence of Xingxihu Reservoir shows a downward trend, the predicted sequence of Mulanghe Reservoir shows an upward trend, and there is no significant change trend in Weishanhu Reservoir. The stationarity of the predicted sequence is consistent with that of the original sequence, indicating that the ARIMA model’s prediction results have a certain degree of accuracy. Based on the predicted sequences, it can be found that the variation range of carcinogenic risk for the three drinking water sources in the following year is 1.0–1.9 × 10^−4^ a^−1^ for Mulanghe Reservoir, 4.6–6.2 × 10^−5^ a^−1^ for Xingxihu Reservoir, and 1.4–2.4 × 10^−4^ a^−1^ for Weishanhu Reservoir. The averages were 1.6 ×10^−4^ a^−1^ for Mulanghe Reservoir, 5.4 × 10^−5^ a^−1^ for Xingxihu Reservoir, and 1.6 × 10^−4^ a^−1^ for Weishanhu Reservoir, all exceeding the maximum acceptable risk level of 1.0 × 10^−6^ a^−1^ recommended by the Swedish Environmental Protection Agency, the Royal Society of the United Kingdom, and the Dutch Ministry of Infrastructure and Water Management. Therefore, in accordance with the principle of health risk management based on maximum expected loss, the carcinogenic risk of Cr(VI) intake in children should be given particular attention in future heavy metal risk management work.

### 3.5. Limitations and Uncertainty

Several limitations and sources of uncertainty should be acknowledged in this study. First, the health-risk assessment was quantified primarily based on the ingestion (direct drinking) pathway. Although ingestion is widely recognized as the dominant exposure route for drinking-water contaminants and dermal contact and inhalation generally contribute much less to overall risk [61], excluding these pathways may still lead to a slightly conservative estimation and does not account for potential additive risks from multiple exposure routes.

Second, the carcinogenic risk assessment was calculated solely based on Cr(VI). Other potentially carcinogenic metals/metalloids (e.g., As, Cd, and Pb) were included in the monitoring program but remained below the method detection limits during the study period, and thus were not incorporated into quantitative carcinogenic risk calculation. Accordingly, the reported carcinogenic risk likely represents a lower-bound estimate of the overall carcinogenic risk under the current monitoring conditions.

Third, the dataset was derived from monthly monitoring of three centralized drinking-water reservoirs in Xingyi City during 2021–2023. Accordingly, the findings may not fully represent other water sources in the region or conditions outside the investigated scope. In addition, ARIMA-based forecasting was conducted only for the next year. Such short-term prediction assumes that temporal patterns remain relatively stable and may not capture long-term trends or abrupt changes driven by extreme rainfall events, sudden pollution inputs, or shifts in water management practices. The prediction results should thus be interpreted as short-term early-warning information under current conditions rather than long-term deterministic projections.

Finally, the results of this study are specific to Southwest Guizhou, where hydrogeochemical conditions are strongly influenced by karst geology and associated water–rock interactions. Therefore, the dominant risk drivers identified here (e.g., Cr(VI)-driven carcinogenic risk) may not be directly transferable to regions with different geological backgrounds, land-use patterns, or pollution sources. Future studies are recommended to expand monitoring coverage to additional water sources, incorporate multi-pathway exposure assessment where appropriate, and integrate source apportionment and scenario-based modeling to further improve risk characterization and prediction.

## 4. Conclusions

This study conducted long-term monitoring and health-risk assessment of eight heavy metals (Ba, V, Cr(VI), Mn, Mo, Ni, Cu, and Fe) in three centralized drinking-water sources in Southwest Guizhou from 2021 to 2023, and further applied ARIMA time-series modeling for short-term risk forecasting. Overall, the concentrations of most target metals complied with relevant standards, while occasional exceedances of Cu and Cr(VI) were observed in certain months.

Health-risk assessment indicated that non-carcinogenic risks for both adults and children remained within acceptable levels, whereas carcinogenic risk was primarily driven by Cr(VI), with children exhibiting higher vulnerability than adults. The ARIMA forecasts suggest that carcinogenic risk associated with Cr(VI) may remain above the commonly accepted benchmark in the near future, highlighting the necessity of continuous monitoring and targeted risk management.

This study provides scientific evidence for risk-based management and early warning of drinking-water safety in karst regions. Nevertheless, several limitations should be acknowledged. The findings are specific to the investigated reservoirs and study period, and the carcinogenic risk assessment was mainly based on Cr(VI) because other carcinogenic elements were below detection limits. In addition, ARIMA predictions were limited to one year ahead and are subject to uncertainty under changing environmental conditions. Future research should expand monitoring coverage to additional water sources, integrate multi-pathway exposure assessment where appropriate, and incorporate source apportionment and scenario-based modeling to further strengthen risk characterization.

## Figures and Tables

**Table 1:** Method detection limits (MDLs) of trace metals/metalloids analyzed in this study (μg/L).

Analyte	Cu	Zn	Se	As	Hg	Cd	Cr(VI)	Pb	Fe	Mn	Mo	Co	Be	B	Sb	Ni	Ba	V	Tl
MDL	0.08	0.67	0.4	0.3	0.04	0.05	0.2	0.09	0.82	0.12	0.06	0.03	0.04	1.25	0.15	0.06	0.2	0.08	0.02

Note: MDL, method detection limit.

**Table 2:** Analytical methods, instruments, and QA/QC procedures for trace metals/metalloids.

Analyte(s)	Method	Standard	Instrument	QA/QC
Cu, Zn, Cd, Pb, Fe, Mn, Mo, Co, Be, B, Sb, Ni, Ba, V, Tl	ICP–MS	HJ 700-2014	Agilent 7800 ICP–MS (Agilent Technologies, Santa Clara, CA, USA)	External calibration; internal standards; CCV; method blanks; duplicates (10%)
Cr(VI)	UV–Vis (1,5-DPC)	GB 7467-87	UV–Vis spectrophotometer (Shanghai Jinghua Technology Instrument Co., Ltd., Shanghai, China)	Batch calibration; QC standards; blanks; duplicates (10%)
As, Hg, Se	AFS	HJ 694-2014	Atomic fluorescence spectrophotometer (Beijing Jitian Instrument Co., Ltd., Beijing, China)	Batch calibration; QC standards; blanks; duplicates (10%)

Note: ICP–MS, inductively coupled plasma–mass spectrometry; UV–Vis, ultraviolet–visible spectrophotometry; 1,5-DPC, 1,5-diphenylcarbohydrazide; AFS, atomic fluorescence spectrometry; CCV, continuing calibration verification; duplicates (10%) indicate one duplicate sample per 10 routine samples.

**Table 3:** Heavy metal carcinogenicity intensity coefficient and non-carcinogenic reference dose.

Carcinogenic	Cr(VI)	Non-Carcinogenic	Fe	Ba	V	Mo	Cu	Mn	Ni
$Q_{i}$ (kg·d·mg^−1^)	41	$RFD_{i}$ (mg·kg^−1^·d^−1^)	0.3	0.2	0.007	0.005	0.005	0.14	0.84

**Table 4:** Comparison of carcinogenic risks of heavy metals in drinking water and surface water across different regions in China.

Study/Region	Province	Water Body Type	Dominant Carcinogenic Metals	Adult Carcinogenic Risk (×10^−5^ a^−1^)	Child Carcinogenic Risk (×10^−5^ a^−1^)	Child/Adult Risk Ratio
Liu et al. (Chongqing) [19]	Chongqing	Drinking water sources	Cr, As	3.5	8.7	2.5
Wang et al. (Dongjiang River Basin) [4]	Guangdong	Drinking water sources	Cr(VI), As, Pb	0.4–11.4	0.7–21.4	1.8
Chen et al. (Pearl River Estuary) [56]	Guangdong	Surface water	As	1.3–1.0	2.4–1.9	1.8–1.9
Wang et al. (Guiyang) [47]	Guizhou	Drinking water sources	Cr(VI), As, Co, Cd	2.9	4.6	1.6
Liu et al. (Qingjiang River Basin) [7]	Hubei	Surface water	As, Cr, Cd	3.2–5.2	3.7–6.0	1.1
Ren et al. (Weihe River Basin) [53]	Shaanxi	Surface water	Cr, As	0.3	0.4	1.3

**Table 5:** ADF test statistical table.

Data sequences	Difference Order	t	*p*	Critical Value		
				1%	5%	10%
Mulanghe Reservoir	0	−2.0	0.26	−3.6	−2.9	−2.6
	1	−8.4	<0.001			
Xingxihu Reservoir	0	−2.2	0.20	−3.6	−2.9	−2.6
	1	−10.7	<0.001			
Weishanhu Reservoir	0	−4.4	<0.001	−3.6	−2.9	−2.6

**Table 6:** Table of Q statistics of 3 drinking water sources.

	Weishanhu Reservoir		Mulanghe Reservoir		Xingxihu Reservoir	
Item	Statistics	*p* Value	Statistics	*p* Value	Statistics	*p* Value
Q1	2.86	0.09	0.002	0.96	0.26	0.61
Q2	2.95	0.23	0.002	1.00	1.36	0.51
Q3	2.98	0.40	0.03	1.00	1.87	0.60
Q4	4.09	0.39	0.27	0.99	2.41	0.66
Q5	4.09	0.54	0.51	0.99	11.38	0.04
Q6	4.27	0.64	2.69	0.85	12.12	0.06

## Data Availability

The data presented in this study are available on request from the corresponding author. The data are not publicly available due to privacy.
